# Non‐Metal Sulfur Doping of Indium Hydroxide Nanocube for Selectively Photocatalytic Reduction of CO_2_ to CH_4_: A “One Stone Three Birds” Strategy

**DOI:** 10.1002/advs.202401990

**Published:** 2024-06-13

**Authors:** Qinhui Guan, Weiguang Ran, Dapeng Zhang, Wenjuan Li, Na Li, Baibiao Huang, Tingjiang Yan

**Affiliations:** ^1^ College of Chemistry and Chemical Engineering Shaanxi University of Science and Technology Xi'an 710021 P. R. China; ^2^ Key Laboratory of Catalytic Conversion and Clean Energy in Universities of Shandong Province School of Chemistry and Chemical Engineering Qufu Normal University Qufu 273165 P. R. China; ^3^ State Key Laboratory of Crystal Materials Shandong University Jinan 250100 P. R. China

**Keywords:** CO_2_‐to‐CH_4_, indium hydroxide, non‐metal doping, photocatalysis

## Abstract

Photocatalytic CO_2_ reduction is considered as a promising strategy for CO_2_ utilization and producing renewable energy, however, it remains challenge in the improvement of photocatalytic performance for wide‐band‐gap photocatalyst with controllable product selectivity. Herein, the sulfur‐doped In(OH)_3_ (In(OH)_x_S_y_‐z) nanocubes are developed for selective photocatalytic reduction of CO_2_ to CH_4_ under simulated light irradiation. The CH_4_ yield of the optimal In(OH)_x_S_y_‐1.0 can be enhanced up to 39 times and the CH_4_ selectivity can be regulated as high as 80.75% compared to that of pristine In(OH)_3_. The substitution of sulfur atoms for hydroxyl groups in In(OH)_3_ enhances the visible light absorption capability, and further improves the hydrophilicity behavior, which promotes the H_2_O dissociation into protons (H^*^) and accelerates the dynamic proton‐feeding CO_2_ hydrogenation. In situ DRIFTs and DFT calculation confirm that the non‐metal sulfur sites significantly weaken the over‐potential of the H_2_O oxidation and prevent the formation of ·OH radicals, enabling the stabilization of ^*^CHO intermediates and thus facilitating CH_4_ production. This work highlights the promotion effect of the non‐metal doping engineering on wide‐band‐gap photocatalysts for tailoring the product selectivity in photocatalytic CO_2_ reduction.

## Graphical abstract

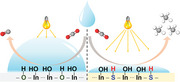

## 1 Introduction

Mimicking natural photosynthesis to achieve the reduction of CO_2_ with H_2_O into value‐added chemicals and fuels has drawn much attention due to the global energy demands and CO_2_ emission reduction.^[^ ^1^ ^]^ In the past years, considerable efforts have been devoted to improving the efficiency of photoreduction of CO_2_ by designing various catalysts, including inorganic TiO_2_,^[^ ^2^ ^]^ Bi_2_WO_6_,^[^ ^3^ ^]^ In_2_O_3_,^[^ ^4^ ^]^ ZnIn_2_S_4_,^[^ ^5^ ^]^ CuIn_5_S_8_,^[^ ^6^ ^]^ CeO_2_,^[^ ^7^ ^]^ graphdiyne (GDY) modified nanocomposite,^[^ ^8^ ^]^ organic g‐C_3_N_4_,^[^ ^9^ ^]^ and covalent organic framework (COF).^[^ ^10^ ^]^ Unfortunately, since the CO_2_ reduction process usually involves the multiple protons coupled electron transfer pathway, the products of CO_2_ photoreduction are complicated, showing a wide variety of distribution, such as carbon monoxide (CO), methanol (CH_3_OH), methane (CH_4_) and even higher hydrocarbons.^[^ ^11^ ^]^ In particular, the conversion of CO_2_ to CH_4_ through the Sabatier reaction has garnered considerable attention because CH_4_ could not only serve as the energy carrier for natural gas but also has a clean combustion characteristic and higher energy density compared with other CO_2_ reduction products.^[^ ^12^ ^]^ Even though the CO_2_‐to‐CH_4_ process is thermodynamically favorable, it is kinetically sluggish since the formation of CH_4_ requires eight electrons and protons and is the most difficult among all the C_1_ products.^[^ ^13^ ^]^

The key to promoting the kinetics of photocatalytic CO_2_ methanation is to enable sufficient photoinduced electrons to participate in the reduction process. Thus, from a viewpoint of kinetic toward CO_2_ methanation, an ideal photocatalyst should have the following prerequisites: good light absorption, proper band structure, and efficient separation of photoinduced carriers.^[^ ^14^ ^]^ Although many narrow‐band‐gap semiconductors show excellent light absorption performance, they usually display low conversion efficiency in CO_2_ photoreduction due to the fast recombination of photogenerated carriers, poor structural stability, and inferior reduction ability of photogenerated electrons. In contrast, some semiconductors with wide‐band gaps can produce photoinduced electrons and holes with sufficiently strong redox capacity, but the limited light absorption severely restricts their photocatalytic performance. Doping of foreign non‐metal elements into wide‐band‐gap semiconductors is a promising and effective strategy to improve the light absorption and separation efficiency of photoinduced carriers, contributing to enhanced photocatalytic performance for CO_2_ reduction.^[^ ^15^ ^]^ In general, the doped non‐metal elements can narrow the band gap by introducing sub‐bands within the original band gap, responsible for improved light absorption and abundant photogenerated electrons. Additionally, the doping states of non‐metal elements usually hybridize with the orbitals from the valence band, rendering that the component, location and the reduction potential of the conduction band remain unchanged.^[^ ^16^ ^]^ What's more, the decreased oxidation capacity upon non‐metal doping can also suppress the formation of strong oxidizing radicals such as ^•^OH and ^•^O_2_^−^, which further reduce the possibility to oxidize the methanation intermediates like *CHO and ^*^CH_3_O, finally leading to the formation of CH_4_ product.^[^ ^17^ ^]^ More recently, many literatures have demonstrated that the introduction of non‐metal elements into inorganic photocatalysts or/and electrocatalysts can promote water dissociation into protons (H^+^) and further deliver the generated H* for CO_2_ reduction.^[^ ^18^ ^]^ As mentioned above, non‐metal‐elements‐doping, as one stone three birds strategy, can significantly increase light absorption and photogenerated electron density, remain strong reduction ability while decreasing the over‐oxidation of the methanation intermediates, and boost water dissociation and proton transfer, thus providing an alternative way to facilitate the photocatalytic CO_2_‐to‐CH_4_ conversion.

Indium hydroxide (In(OH)_3_), an important wide‐band‐gap semiconductor photocatalyst, has received considerable attention in recent years due to its strong redox capacity and excellent surface property.^[^ ^19^ ^]^ Moreover, In(OH)_3_ is a suitable catalyst for CO_2_ photoreduction because of its suitable potentials of the valence band and conduction band.^[^ ^20^ ^]^ However, the wide band gap (5.12 eV) of In(OH)_3_ severely restricts the catalytic efficiency due to the limited light absorption and separation efficiency of photogenerated carriers. To solve these issues, Wang and co‐workers developed vacancy‐engineering strategies (La‐doping or In‐vacancy) to improve the photocatalytic reduction of CO_2_ to produce CO and CH_4_.^[^ ^20^, ^21^ ^]^ Ling et al. further reported a ZnIn_2_S_4_/In(OH)_3‐x_ heterojunction for visible‐light‐driven CO_2_ reduction into CO and found that the constructed type II heterojunction and the defective In(OH)_3‐x_ with frustrated Lewis pairs can synergistically facilitate the photogenerated electron transfer and CO_2_ activation.^[^ ^22^ ^]^ These studies indicate that In(OH)_3_ is a photocatalytic stable and promising catalyst for CO_2_ reduction, however, the designed In(OH)_3_ catalyst meeting the requirements of wide solar absorption and highly active sites for reactants activation remains to be explored and the product selectivity of CO_2_ photoreduction needs to be further improved. The previous work has shown that the partial substitution of HO^−^ by S^2−^ can significantly narrow the band gap of In(OH)_3_ and improve the visible‐light‐driven photocatalytic water splitting and decomposition of gaseous acetone.^[^ ^16^, ^23^ ^]^ Moreover, many studies indicate that the doped sulfur sites play important roles in both H_2_O dissociation and the dynamic migration of protons to produce CHO^*^ intermediates.^[^ ^18^, ^24^ ^]^ A recent study also found that the non‐metal sulfur atom in CuInSnS_4_ could function as the adsorption center for stabilizing the intermediates and thus promoting the photocatalytic conversion of CO_2_ to CH_4_.^[^ ^25^ ^]^ Thus, as mentioned above, we consider that non‐metal sulfur doping of In(OH)_3_ may be a promising photocatalyst toward CO_2_ photoreduction into CH_4_ and it is highly necessary to explore the roles of sulfur sites during photocatalytic process.

Herein, we report a series of S‐doped In(OH)_3_ (denoted as In(OH)_x_S_y_‐*z*) nanocubes with varying S/In atomic ratios for efficient and selective CO_2_ reduction to CH_4_ under UV–vis light irradiation. In the S‐doped In(OH)_3_, the substitution of sulfur atoms for hydroxyl groups rendered the excellent light absorption capacity, improved surface hydrophilicity behavior, and promoted kinetics of H_2_O dissociation as well as proton‐feeding processes, thereby accounting for the significantly enhanced photocatalytic CO_2_ reduction performance. The optimal In(OH)_x_S_y_‐1.0 exhibited an evolution rate of 2.75 µmol g^−1^ h^−1^ and 80.75% product selectivity for CH_4_ production under UV‐vis light irradiation in the absence of any sacrificial agent. The in situ DRIFTS and DFT calculation results demonstrate that the non‐metal sulfur sites play a critical role in promoting H_2_O dissociation into proton (H^+^) and accelerating the dynamic proton‐feeding for the formation of CH_x_^*^ species.

## 2 Results and Discussion

### 2.1 Characterizations of In(OH)_3_ and In(OH)_x_S_y_‐*z*

**Figure 1:**
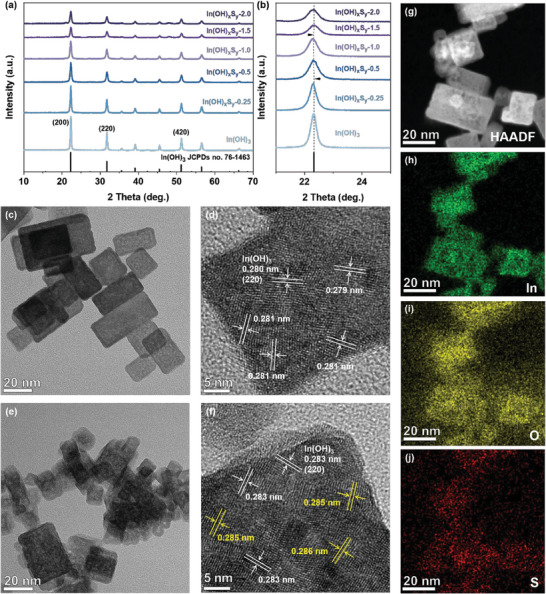
Morphology and structural characterizations. a,b) The XRD patterns of In(OH)_3_ and In(OH)_x_S_y_‐*z* samples. TEM images of c,d) In(OH)_3_ sample and e,f) In(OH)_x_S_y_‐1.0 sample. g–j) EDS element mapping images for In(OH)_x_S_y_‐1.0 sample.

The powder X‐ray diffraction (XRD) patterns of the prepared In(OH)_3_ and In(OH)_x_S_y_‐z samples with different S/In atomic ratios (ranging from 0 to 2) are shown in **Figure** **1**a. All patterns exhibit narrow and sharp diffraction peaks, which match well with the cubic phase of In(OH)_3_ (JCPDS no. 76‐1463). No impurity phases are detected, indicating the well‐preserved In(OH)_3_ phase structure after the sulfurization process under the hydrothermal conditions. As the S/In atomic ratio increased from 0 to 1, S^2−^ began to partially substitute for OH^−^, resulting in a gradual and slight shift in the diffraction peaks toward the lower angle (Figure 1b). Additionally, the diffraction peaks are noticeably weakened and broadened, suggesting partial damage to the crystal structure and a decrease in crystallinity. However, when the S/In atomic ratio exceeds 1, the diffraction peaks do not shift further. This phenomenon indicates a homogeneous incorporation of S^2−^ into the In(OH)_3_ lattice, resulting in the formation of the In(OH)_x_S_y_‐*z* samples.^[^ ^16b^ ^]^ The average crystallite sizes calculated from the *Debye–Scherrer* equation indicate that the size of the series of In(OH)_x_S_y_‐*z* samples formed by substituting OH^−^ with S^2−^ gradually decreases, while the specific surface area and pore size increase first and then decrease (Figure S1 and Table S1, Supporting Information). When the atomic ratio of S/In is 1.0, the resulting In(OH)_x_S_y_‐1.0 sample exhibits a moderate average crystallite size of 19.88 nm, the highest BET surface area of 69.71 m^2^ g^−1^, and the largest mean pore size of 3.09 nm. Transmission electron microscope (TEM) images of the pure In(OH)_3_ and In(OH)_x_S_y_‐1.0 samples are presented in Figure 1c,e, respectively. It is observed that both samples exhibit a cubic structure with side length between 10 and 60 nm. Note that the In(OH)_x_S_y_‐1.0 sample shows a decreased size and plenty of mesopores within the nanocubes, which should be primarily attributed to the bubbling effect of generated CO_2_ from thiourea decomposition (Figure S2, Supporting Information). The HRTEM images (Figure 1d,f) reveal that the lattice fringes of In(OH)_3_ and In(OH)_x_S_y_‐1.0 sample display interplanar spacings of 0.280–0.286 nm, corresponding to the (220) crystal plane of cubic In(OH)_3_, indicating that the lattice spacing expands slightly with the substitution of S^2−^. These observations fit well with the XRD results. The reduced particle sizes and the improved specific surface area for the In(OH)_x_S_y_‐1.0 sample can provide more active sites, leading to potential improvement in photocatalytic performance. In addition, the energy dispersive spectrometer (EDS) element analysis of the In(OH)_x_S_y_‐1.0 sample (Figure 1g–j) confirms the homogeneous distribution of In, O, and S elements.

**Figure 2:**
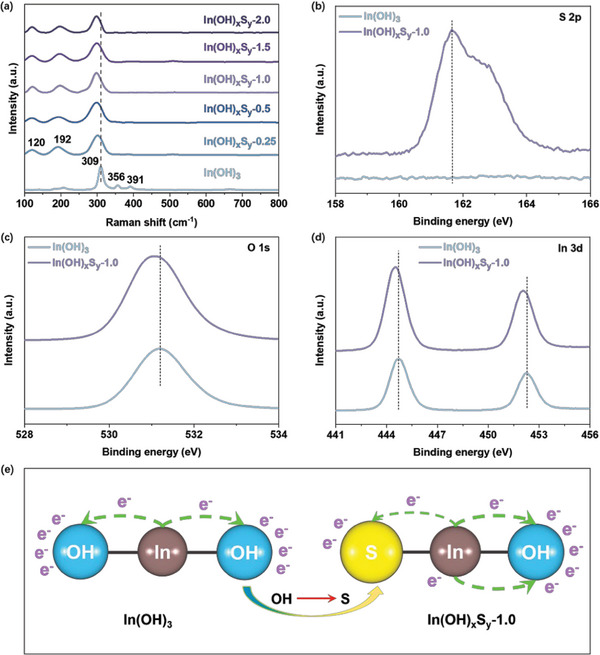
Geometric and electronic structures. a) Raman spectra of In(OH)_3_ and In(OH)_x_S_y_‐*z* samples. High resolution XPS of b) S 2p, c) O 1s and d) In 3d of In(OH)_3_ sample and In(OH)_x_S_y_‐1.0 sample. e) Schemes of electron transfer in In(OH)_3_ (left) and In(OH)_x_S_y_‐1.0 (right), respectively.

Raman spectra were further conducted to confirm the S^2−^ doping into In(OH)_3_. As shown in **Figure** **2**a, the peaks observed at 309, 356 and 391 cm^−1^ of bare In(OH)_3_ correspond to the phonon vibration modes of cubic In(OH)_3_.^[^ ^20^, ^26^ ^]^ In the In(OH)_x_S_y_‐*z* samples, the main peak ≈309 cm^−1^ exhibits an obvious blue shift, while the other peaks related to In─O bond disappear. These changes can be attributed to the disruption of the crystal symmetry of In(OH)_3_ caused by the introduction of S^2−^.^[^ ^27^ ^]^ Additionally, two peaks associated with the In─S bond at 120 and 192 cm^−1^ are also observed,^[^ ^28^ ^]^ further confirming the successful incorporation of S^2−^ into the In(OH)_3_ structure.

To preliminarily explore the influence of S^2−^ doping on the electronic structure of In(OH)_x_S_y_‐*z* samples, XPS analysis was conducted. All the spectra were calibrated using the C 1s reference of 284.8 eV. As shown in Figure 2b, the characteristic XPS peak of S^2−^ can be well detected on In(OH)_x_S_y_‐1.0. Moreover, it is noteworthy that, upon introducing S^2−^, the O 1s and In 3d of In(OH)_x_S_y_‐1.0 shift toward lower binding energies (Figure 2c,d), indicating an enhancement in their electron density. Since S^2−^ has a weaker electronegativity than OH^−^, the newly introduced S^2−^ exhibits a lower electron adsorption capability on the adjacent In atom compared to the O atom/OH group on the other side of the In atom. This enables the hydroxyl group to acquire more electrons from the In atom, consequently breaking the symmetric OH‐In‐OH active centers and constructing S‐In‐OH asymmetric electron enrichment active sites (Figure 2e), which thereby can promote the adsorption and activation of reactant molecules. The actual content of S in the In(OH)_x_S_y_ can be determined to be ≈5.06% (Table S2, Supporting Information). The electron paramagnetic resonance spectroscopy (EPR) excludes the possibility of the formation of oxygen vacancies after sulfur atom substitution in In(OH)_x_S_y_‐*z* samples (Figure S3, Supporting Information).

**Figure 3:**
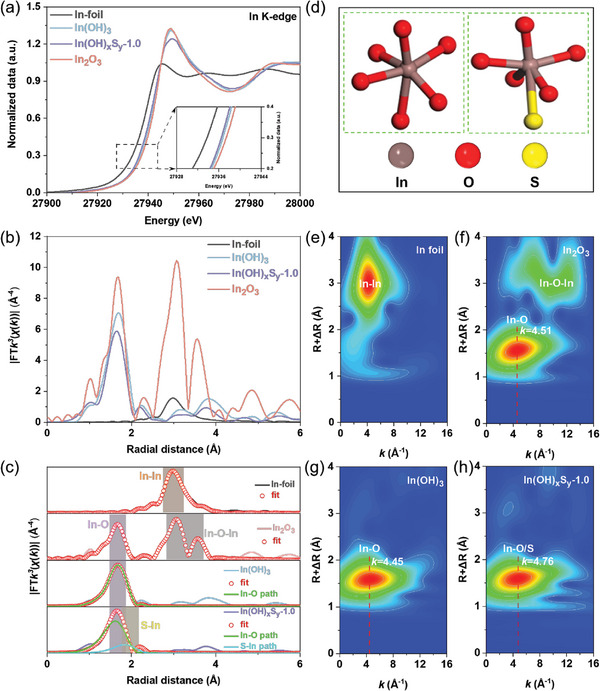
Synchrotron radiation X‐ray absorption spectroscopy. a) Normalized In K‐edge XANES spectra, b) *k* ^3^‐weighted In K‐edge Fourier‐transform EXAFS spectra, c) EXAFS fitting curves of In(OH)_3_ and In(OH)_x_S_y_‐1.0, as well as In foil and In_2_O_3_ references. d) The structure model of In(OH)_3_ (left) and In(OH)_x_S_y_‐1.0 (right). e–h) WT for the *k* ^3^‐weighted EXAFS signal at In K‐edge of In foil, In_2_O_3_, In(OH)_3_, and In(OH)_x_S_y_‐1.0, respectively.

Synchrotron radiation X‐ray absorption spectroscopy (XAS) was further used to obtain the information of the chemical environment of In atom in the structure of In(OH)_3_ and In(OH)_x_S_y_‐1.0.^[^ ^29^ ^]^ The In K‐edge X‐ray absorption near‐edge structure (XANES) spectra in **Figure** **3**a reveal that the adsorption edge of In(OH)_x_S_y_‐1.0 is slightly lower than that of In(OH)_3_. This distinction can be attributed to the formation of the In–S coordination structures, where electronegativity of S is lower compared to that of O, leading to an increased electron density of In atom and a blue shift of the In K‐edge of the absorption. The Fourier‐transformed In K‐edge extended X‐ray absorption fine structure (EXAFS) spectra are shown in Figure 3b. The peak intensity and position of In(OH)_x_S_y_‐1.0 are lower and shifted toward lower radial distances compared to the In(OH)_3_, indicating fewer In─O coordination numbers and shorter In─O bond distances in In(OH)_x_S_y_‐1.0. This could be attributed to the presence of the In─S structures in In(OH)_x_S_y_‐1.0, leading to a reduction in the overall In─O coordination numbers and shorter In─O bond distances.^[^ ^30^ ^]^ Furthermore, EXAFS fitting analysis (Figure 3c; Table S3, Supporting Information) reveals that In(OH)_3_ conforms to hexa‐coordinated In─O, while In(OH)_x_S_y_‐1.0 exhibits penta‐coordinated In─O and one In─S coordination (Figure 3d).^[^ ^31^ ^]^ Meanwhile, the coordination bond structures of the In atom in In(OH)_3_ and In(OH)_x_S_y_‐1.0 were vividly determined through wavelet transform (WT) (Figure 3e–h). It is observed that the wave center marked by red dash line of In(OH)_x_S_y_‐1.0 (*k* = 4.75 Å^−^¹) is slightly shifted toward higher values than that of In(OH)_3_ (*k* = 4.45 Å^−^¹). This shift can be attributed to the coordination of In with S atoms, which possess a higher atomic number than O atoms, consequently inducing an overall high position shift in the wave peaks.

**Figure 4:**
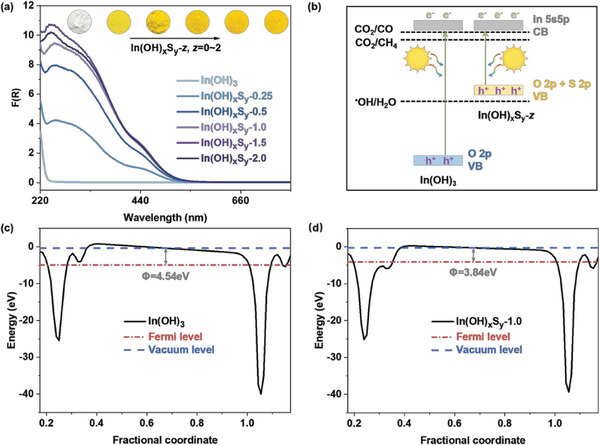
Optical property and band structures. a) UV–vis DRS spectra of In(OH)_3_ and In(OH)_x_S_y_‐*z* samples. b) Bandgap diagram of In(OH)_3_ and In(OH)_x_S_y_‐*z* samples. The work function of c) In(OH)_3_ and d) In(OH)_x_S_y_‐1.0.

The UV–vis diffuse reflectance spectra (DRS) of In(OH)_3_ and In(OH)_x_S_y_‐*z* samples are shown in **Figure** **4**a. It is shown that the white In(OH)_3_ sample can only absorb UV light below 250 nm. In contrast, all the yellow In(OH)_x_S_y_‐*z* samples exhibit considerable absorption in both the UV and visible light region (up to 520 nm), indicating that the substitution of S^2−^ effectively modifies the energy structure and broadens the absorption edge of wide‐band‐gap In(OH)_3_. The band gap of the In(OH)_x_S_y_‐*z* samples narrows to ca. 2.1 eV (Figure S4, Supporting Information). According to Mott–Schottky analysis (Figure S5, Supporting Information), the electronic band structure diagrams reveal that S^2−^ substitution notably reduces the oxidation potential with an upper shift of the valence band, while maintains the substantial reduction potential for the In(OH)_x_S_y_‐*z* samples (Figure S6, Supporting Information). Density of states (DOS) analysis demonstrates that both In(OH)_3_ and In(OH)_x_S_y_‐1.0 consist of the same In 5s5p orbital for the conduction band (CB) (Figure S7a,b, Supporting Information). In the case of valence band (VB), In(OH)_3_ is comprised of the O 2p orbital, while In(OH)_x_S_y_‐1.0 is mainly composed of the hybridization of S 2p and O 2p orbitals. Thus, the incorporation of S^2−^ leads to the formation of a new hybridized state, resulting in an upward shift of the VB energy levels (Figure 4b). The work function (W_F_) refers to the minimum energy required for an electron to transition from the Fermi level to the vacuum level. Through the substitution of S^2−^, the calculated W_F_ of In(OH)_x_S_y_‐1.0 is 3.84 eV, which is 0.70 eV lower than that of In(OH)_3_ (4.54 eV) (Figure 4c,d). This indicates that the substitution of S^2−^ increases the escape of surface electrons and thereby provides more charge carriers to participate in the photocatalytic reaction.^[^ ^32^ ^]^

### 2.2 Photocatalytic CO_2_ Reduction Performance

**Figure 5:**
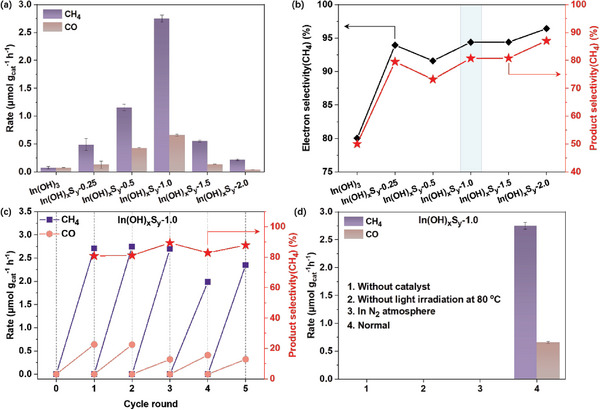
Photocatalytic CO_2_ reduction performances. a) Production rate of CH_4_ and CO. b) Product selectivity and electron selectivity of CH_4_. c) Stability test for the In(OH)_x_S_y_‐1.0 sample. d) Control experiments.

The photocatalytic CO_2_ reduction performance was evaluated in a custom‐made bipass glass reaction system under simulated solar light irradiation in the absence of any sacrificial agent (Figure S8, Supporting Information). As shown in **Figure** **5**a, In(OH)_3_ could generate CH_4_ and CO with a similar production rate of 0.07 µmol g^−1^ h^−1^. In contrast, all the In(OH)_x_S_y_‐*z* samples showed improved activity for the formation of CH_4_ and CO. It is notable that the CH_4_ and CO evolution rates initially increased and then gradually decreased as the S‐doping content varied from an atomic ratio of S/In = 0.25 to S/In = 2.0. Among the tested In(OH)_x_S_y_‐*z* samples, In(OH)_x_S_y_‐1.0 displayed the highest production rate of 2.75 µmol g^−1^ h^−1^ for CH_4_ and 0.65 µmol g^−1^ h^−1^ for CO, which were 39 and 9 times higher than that of In(OH)_3_, respectively. The volcano‐type activity trend of In(OH)_x_S_y_‐*z* should be ascribed to the changes in the textural structures, electronic structures and surface active sites. In(OH)_x_S_y_‐*z* samples, with enhanced optical absorption properties and appropriate electronic band structure, could also produce H_2_ with an identical volcano‐type trend (Figure S9, Supporting Information). Compared to pristine In(OH)_3_, it is noted that all the In(OH)_x_S_y_‐*z* samples exhibited not only an improved production rate but also higher CH_4_ selectivity both in electron selectivity and product selectivity (Figure 5b). This was evidenced by an increase in electron selectivity from 91.61% to 96.41%, and product selectivity from 73.18% to 87.04% for In(OH)_x_S_y_‐*z* samples. Particularly for the In(OH)_x_S_y_‐1.0, which had the highest CH_4_ production rate among the In(OH)_x_S_y_‐*z* samples, the electron selectivity and product selectivity of CH_4_ were 94.38% and 80.75%, respectively. The remarkable enhancement in CH_4_ production of In(OH)_x_S_y_‐1.0 could be primarily attributed to the synergetic effects of its textural and electronic structures. Collected from the characteristic results, the In(OH)_x_S_y_‐1.0 sample shows the largest specific surface area, the most negative reduction potential and reduced oxidation potential, and the most negative surface electronegativity. As compared with the recently reported In(OH)_3_‐based photocatalysts, such In(OH)_x_S_y_‐1.0 catalyst exhibits considerably excellent photocatalytic performance toward the photoreduction of CO_2_ into CH_4_ (Table S4, Supporting Information). Moreover, a minimal and unquantifiable O_2_ could be detected as oxidative product for In(OH)_x_S_y_‐*z* samples due to the absence of any sacrificial agent (Figure S10, Supporting Information). Stability tests were conducted on the In(OH)_x_S_y_‐1.0 sample for cycle tests, as shown in Figure 5c. The results indicate that after five successive tests, the In(OH)_x_S_y_‐1.0 sample could still maintain to an acceptable CH_4_ product rate of 2.35 µmol g^−1^ h^−1^ and CO product rate of 0.32 µmol g^−1^ h^−1^, with a good CH_4_ selectivity of 87.99%, suggesting an excellent photocatalytic stability for CO_2_ reduction. Moreover, the spent‐In(OH)_x_S_y_‐1.0 after the successive cycle tests showed no significant changes in crystal phase, chemical state, chemical composition and morphology, confirming its structural stability (Figure S11, Supporting Information). Additionally, to substantiate that the production of CH_4_ and CO was inseparable from the photocatalytic reaction with catalyst and the injected raw gas CO_2_ participated in, several parallel control experiments were conducted on the In(OH)_x_S_y_‐1.0 sample. Figure 5d demonstrates that no product was observed in the absence of catalyst, without light irradiation but with heat or in N_2_ atmosphere, strongly confirming that both carbon products were exclusively derived from photocatalytic reduction of CO_2_ with In(OH)_x_S_y_‐1.0 as catalyst.

### 2.3 Interfacial Charge Transfer and Separation

**Figure 6:**
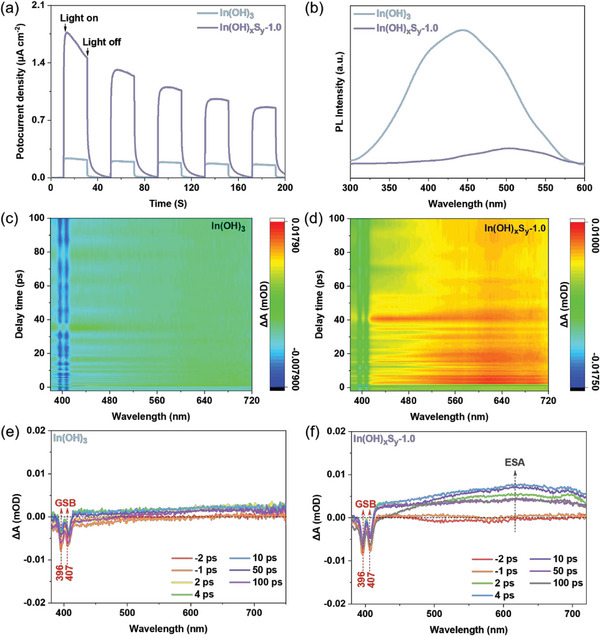
The dynamics of charge carrier transfer. a) Transient photocurrent response and b) Room‐temperature PL spectrum of In(OH)_3_ and In(OH)_x_S_y_‐1.0 samples. c) fs‐TA spectra of In(OH)_3_, d) fs‐TA spectra of In(OH)_x_S_y_‐1.0, e) fs‐TA spectra at seven different time scales of In(OH)_3_, f) fs‐TA spectra at seven different time scales of In(OH)_x_S_y_‐1.0.

To comprehensively investigate the interfacial charge kinetics of both the pristine In(OH)_3_ and the In(OH)_x_S_y_‐1.0 sample, a series of photochemical and photo‐electrocatalytic measurements were performed. **Figures** **6**a and S12a (Supporting Information) demonstrate that In(OH)_x_S_y_‐1.0 exhibited higher photocurrent density with a three times enhancement and lower impedance compared to that of In(OH)_3_, indicating a better interfacial charge separation and transfer efficiency.^[^ ^33^ ^]^ The CV curve area is determined by the amount of charge transferred during the electrode scan, with a larger charge indicating a stronger charge transfer ability.^[^ ^21^ ^]^ The result demonstrates that compared to the In(OH)_3_ sample (0.03 mC), the In(OH)_x_S_y_‐1.0 sample showed a higher CV curve area (0.05 mC) (Figure S12b, Supporting Information), revealing a faster electron transfer rate, which further proved the superior charge separation and transfer of In(OH)_x_S_y_‐1.0. The LSV curves carried out under different conditions including in N_2_‐saturated atmosphere, in CO_2_‐saturated atmosphere and in CO_2_‐saturated atmosphere with light irradiation were illustrated in Figure S12c (Supporting Information). The results show that both the polarization curves of In(OH)_3_ and In(OH)_x_S_y_‐1.0 exhibited higher current density when N_2_‐saturated atmosphere was replaced by CO_2_‐saturated atmosphere, revealing that these two samples were active for CO_2_ conversion.^[^ ^34^ ^]^ It is also noted that the current density in the CO_2_‐saturated atmosphere for In(OH)_x_S_y_‐1.0 is much higher than that of In(OH)_3_, implying that In(OH)_x_S_y_‐1.0 has better CO_2_ reduction performance. Furthermore, when light irradiation was introduced into the CO_2_‐saturated atmosphere, the current density of In(OH)_x_S_y_‐1.0 is further enhanced, far exceeding that of In(OH)_3_. This enhanced performance can be attributed to the benefited light absorption and improved photo‐generated charges after S doping. The room‐temperature photoluminescence (PL) spectra of In(OH)_3_ and In(OH)_x_S_y_‐1.0 are shown in Figure 6b. The pristine In(OH)_3_ exhibited a strong green emission ≈444 nm, while the incorporation of S^2−^ into In(OH)_3_ leads to a weakening and broadening PL emission peak, indicating an enhanced separation efficiencies of the photogenerated electron–hole pairs in the In(OH)_x_S_y_‐1.0.^[^ ^35^ ^]^ Therefore, the accrued results of photocurrent, CV, LSV, and PL have consistently confirmed that the S doping of In(OH)_3_ can accelerate the electron transfer rate and thereby promote the separation and transfer of charge carriers.

Ultra‐fast femtosecond time‐resolved transient absorption (fs‐TA) spectroscopy measurements were systematically conducted using a 290 nm pump pulse within the range of −2–100 ps to investigate the charge carrier dynamics and the lifetime of the excited photoelectrons in In(OH)_3_ and In(OH)_x_S_y_‐1.0 samples. In the fs‐TA spectra of In(OH)_3_ (Figure 6c,e), two negative ground‐state bleaching (GSB) signals at and 396 and 407 nm were detected, which generally originate from the electrons transition from VB to CB.^[^ ^36^ ^]^ The fs‐TA spectra of In(OH)_x_S_y_‐1.0 (Figure 6d,f) also revealed two intensified negative GSB signals at 396 and 407 nm. This enhancement can be ascribed to the easier and effective electrons transition from the VB to the CB in the reduced bandgap caused by S^2−^ substitution. Moreover, the In(OH)_x_S_y_‐1.0 sample exhibited a series of prolonged excited‐state absorption (ESA) signals, which can be attributed to the electrons transition from lower energy levels to higher energy levels within the CB.^[^ ^36a^ ^]^ The detection of additional ESA signals in In(OH)_x_S_y_‐1.0 indicates a greater propensity for generating free and shallowly trapped electrons during the pump light process. These electrons are recognized as the active species responsible for driving the photocatalytic reduction reaction.^[^ ^37^ ^]^ To further reveal the excited state kinetics of In(OH)_3_ and In(OH)_x_S_y_‐1.0, the fs‐TA kinetic decay plots and the corresponding fitting curves at 396 and 407 nm probe light are compared. All fs‐TA kinetic plots can be fitted using a two‐exponential decay function. The average decay lifetime at 396 nm is 0.43 ps and 1.22 ps for In(OH)_3_ and In(OH)_x_S_y_‐1.0, respectively (Figure S13a, Supporting Information), while that at 407 nm is 0.30 ps and 0.94 ps, respectively (Figure S13b, Supporting Information). The fs‐TA kinetic decay lifetime results demonstrate that appropriate S atom doping can prolong the GSB decay lifetime and significantly inhibit the recombination of photogenerated electrons and holes, which is favorable for promoting photocatalytic reduction.^[^ ^38^ ^]^

**Figure 7:**
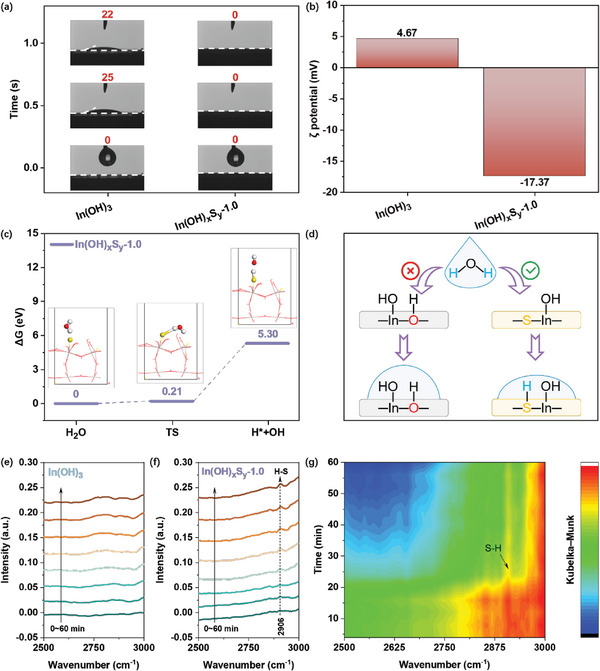
The adsorption and activation of H_2_O molecules. a) The water contact angles; b) ζ potentials diagrams. c) DFT‐calculated Gibbs free energy diagrams of H_2_O activation and dissociation on the surfaces of In(OH)_x_S_y_‐1.0. The red, yellow and white atoms represent oxygen, sulfur and hydrogen atoms, respectively. d) Schematic diagram of the adsorption and activation of In(OH)_3_ (left) and In(OH)_x_S_y_‐1.0 (right) for water. In situ DRIFTS spectra of CO_2_ and H_2_O vapor co‐adsorbed on e) In(OH)_3_ and f) In(OH)_x_S_y_‐1.0. g) Corresponding 2D color‐filled contour plot of in situ DRIFTS spectra of CO_2_ and H_2_O vapor co‐adsorbed on In(OH)_x_S_y_‐1.0.

It has been demonstrated that the hydrophilicity of semiconductor photocatalysts plays a critical role in photocatalytic CO_2_ with water.^[^ ^3^ ^]^ Therefore, to further understand the effect of S doping on the surface hydrophilicity of the catalysts, a series of testing methods exploring the surface properties of the catalyst were employed. **Figure** **7**a shows the water contact angle measurements of the In(OH)_3_ and In(OH)_x_S_y_‐1.0 samples. The pristine In(OH)_3_ exhibited a contact angle of 25° after 0.5 s and 22° after 1.0 s. In contrast, the In(OH)_x_S_y_‐1.0 showed an improved hydrophilic property as the contact angle decreased to 0° once water was dropped on the catalyst surface. Furthermore, the ζ potentials revealed that the In(OH)_x_S_y_‐1.0 exhibited an polar surface with a negatively charge of ‐17.37 mV due to the S‐In‐OH asymmetric active canters, whereas the In(OH)_3_ displayed a positive charge surface of 4.67 mV (Figure 7b). The negatively charged surface of In(OH)_x_S_y_‐1.0 can establish strong electrostatic interactions with H^+^ in water, resulting in a better wettability.^[^ ^1^, ^39^ ^]^ The H_2_O adsorption and activation energy on the crystal (200) plane of In(OH)_3_ and In(OH)_x_S_y_‐1.0 was further calculated. For the In(OH)_3_ sample, the theoretical modelling illustrates that the In(OH)_3_ sample itself cannot adsorb and activate H_2_O molecules. Thus, in the photocatalytic reduction process, the required protons can only be generated from the spontaneous dissociation of water molecules with an energy barrier of 8.14 eV (Figure S14, Supporting Information). As for In(OH)_x_S_y_‐1.0, it is observed that the adsorbed H_2_O undergoes a kinetic process to form a transition state (TS) at S^2−^ site, and then dissociates into ^*^H and ^*^OH with a significantly reduced energy barrier of 5.30 eV (Figure 7c). Therefore, the S‐In‐OH asymmetric active canters promote the H_2_O dissociation and could provide more protons to feed the CO_2_ reduction (Figure 7d). In situ diffuse reflectance infrared Fourier transform spectroscopy (in situ DRIFTS) was further performed to provide evidence for the formation of S─H bond. A new peak at 2906 cm^−1^ was clearly observed on the surface of In(OH)_x_S_y_‐1.0, indicating the formation of the S─H bond/site on In(OH)_x_S_y_‐1.0 during H_2_O adsorption (Figure 6e–g).^[^ ^40^ ^]^ These results further reinforce the role of introduced S^2−^ as a vital active site in In(OH)_x_S_y_‐1.0, which not only facilitates the light absorption and charge separation efficiency, but also enhances the hydrophilicity, resulting in the improved adsorption and activation of H_2_O.^[^ ^18a^ ^]^

In the heterogeneous catalytic process, CO_2_ adsorption is also considered as a pivotal factor determining the catalytic efficiency and product selectivity. The CO_2_ adsorption isotherms of In(OH)_3_ and In(OH)_x_S_y_‐1.0 were investigated to obtain the adsorption ability of CO_2_ between them. As shown in Figure S15a (Supporting Information), In(OH)_x_S_y_‐1.0 exhibited an adsorption capacity of 3.65 cm^3^ g^−1^, which is 1.73 times higher than that of the In(OH)_3_ (2.11 cm^3^ g^−1^). The increased CO_2_ adsorption capacity observed in In(OH)_x_S_y_‐1.0 can be attributed to the strong interaction between acidic CO_2_ and the electron‐enriched hydroxyl groups in S‐In‐OH that can function as Lewis basic sites. In addition, the increased specific surface area and porous structure of In(OH)_x_S_y_‐1.0 can also contribute to the improved CO_2_ adsorption. Since CO is an important intermediate in the generation of CH_4_, the interaction between CO and the catalyst was further performed by CO temperature‐programmed desorption (CO‐TPD). The results demonstrate a substantial increase in the desorption temperature of CO on In(OH)_x_S_y_‐1.0 compared to In(OH)_3_, rising from 65 °C to ≈198 °C, as illustrated in Figure S15b (Supporting Information). This implies that In(OH)_x_S_y_‐1.0 has the stronger ability to bind with CO and will lead more CO participate in the subsequent deep hydrogenation reaction to generate CH_4_.

### 2.4 Insights into the Photocatalytic CO_2_ Reduction Mechanism

**Figure 8:**
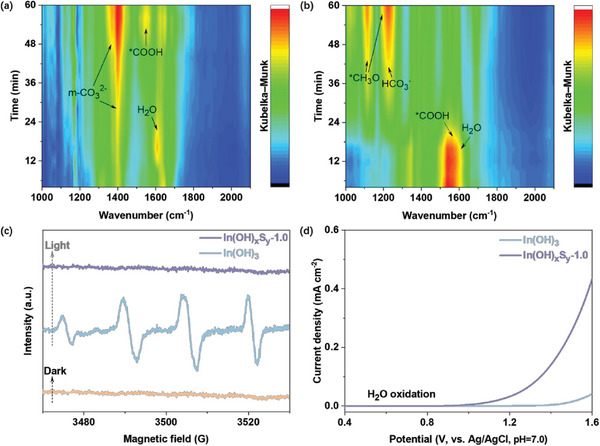
The adsorption and activation of CO_2_ molecules. 2D color‐filled contour plots of in situ DRIFTS spectra for a) In(OH)_3_ and b) In(OH)_x_S_y_‐1.0. c) DMPO spin‐trapping EPR spectra recorded for ^•^OH with and without light irradiation, and d) LSV curves of In(OH)_3_ and In(OH)_x_S_y_‐1.0 samples.

In order to understand the photocatalytic pathway for CO_2_ reduction with water on In(OH)_3_ and In(OH)_x_S_y_‐1.0, in situ DRIFTS measurements were performed under reaction operando conditions. As shown in **Figure** **8**a,b, the peak ≈1620 cm^−1^ for both In(OH)_3_ and In(OH)_x_S_y_‐1.0 samples can be related to the bending vibration of H_2_O.^[^ ^41^ ^]^ As the reaction proceeded, the intensity of the H_2_O peak gradually decreased and finally disappeared, indicating that the adsorbed H_2_O was participated in the reaction process. It is also noted that the intensity of H_2_O peak on In(OH)_x_S_y_‐1.0 is much higher than that on In(OH)_3_, reflecting the strong adsorption ability of H_2_O over In(OH)_x_S_y_‐1.0 with non‐metal sulfur active sites. Apart from H_2_O, the evolution of surface adsorbed species over these two samples is quite distinct. In the case of In(OH)_3_, monodentate carbonate (m‐CO_3_^2−^) at ≈1370 and 1393 cm^−1^ can be clearly observed as the main intermediate species during the photocatalytic process.^[^ ^21^, ^42^ ^]^ However, for the In(OH)_x_S_y_‐1.0 sample, the formate (^*^COOH) at 1551 cm^−1^ can be obviously seen and gradually decreased as the reaction proceeded.^[^ ^43^ ^]^ Simultaneously, two signals that are assigned to bicarbonate (HCO_3_^−^) located at 1224 cm^−1^ and methoxy (^*^CH_3_O) at 1114 and 1184 cm^−1^ appeared.^[^ ^3^, ^6^ ^]^ The formation of HCO_3_^−^ and ^*^CH_3_O over In(OH)_x_S_y_‐1.0 reveals the deep hydrogenation of the intermediates by doped S sites. As well known, both HCO_3_^−^ and ^*^CH_3_O play important roles as the intermediate species in the photocatalytic CO_2_‐to‐CH_4_ conversion.^[^ ^3^, ^7^ ^]^ On the basis of the in situ DRIFTS results, it is speculated that the CO_2_ photoreduction to CH_4_ on In(OH)_x_S_y_‐1.0 could proceed via a CO_2_ → ^*^COOH → ^*^CO → ^*^CHO → ^*^CH_3_O → ^*^CH_3_ → ^*^CH_4_ → CH_4_ path.^[^ ^43^, ^44^ ^]^ Xu et al. elucidated the photocatalytic reduction of CO_2_ to CH_4_ through the formation of key intermediate species including ^*^COOH, ^*^CO, ^*^CHO, and ^*^CH_3_O.^[^ ^45^ ^]^ The pivotal role of these aforementioned species in facilitating the CO_2_‐to‐CH_4_ conversion process has also been reported by Xie et al.^[^ ^6^ ^]^

According to the diagram of electronic band structure, the standard oxidation/reduction potential of ^•^OH/H_2_O is 2.27 eV, while the VB oxidation potentials of In(OH)_x_S_y_‐*z* samples are lower than this value, suggesting that the remaining holes cannot oxidize OH^−^ or H_2_O to form active ^•^OH species theoretically.^[^ ^46^ ^]^ DMPO spin‐trapping electron paramagnetic resonance (EPR) results demonstrate that compared to In(OH)_3_, In(OH)_x_S_y_‐1.0 sample exhibits no ^•^OH signal under light irradiation (Figure 8c), indicating that In(OH)_x_S_y_‐1.0 can effectively suppress the generation of ^•^OH. Inhibition of ^•^OH generation is beneficial for catalysing CO_2_ reduction, because ^•^OH can oxidize reaction intermediates, such as ^*^CHO and ^*^CH_3_O that are generated during photocatalytic CO_2_ reduction.^[^ ^17^ ^]^ By comparing the oxidation potentials of water, it was found that the inhibition of ^•^OH formation over In(OH)_x_S_y_‐1.0 is achieved by significantly reducing the over‐potential for H_2_O oxidation (Figure 8d).

**Figure 9:**
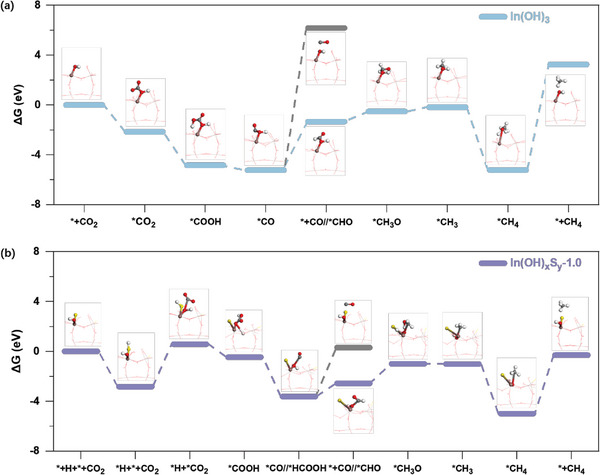
Gibbs free energy diagrams of reaction pathways. Photocatalytic CO_2_ reduction on the surfaces of a) In(OH)_3_ and b) In(OH)_x_S_y_‐1.0. Insets are the corresponding structures of reaction intermediates.

To corroborate these experimental observations, theoretical models were developed to investigate the impact of introduced S^2−^ on the CO_2_ reduction process. These models were constructed on the crystal (200) plane of both the In(OH)_3_ and the In(OH)_x_S_y_‐1.0. **Figure** **9**a,b shows the step‐by‐step Gibbs free energy diagrams, illustrating the process of CO_2_ reduction to CH_4_. All intermediate states were meticulously optimized by considering a range of potential pathways to achieve the lowest energy state. First, both the In(OH)_3_ and In(OH)_x_S_y_‐1.0 require lower energy for the exothermic process (−1.98 eV for In(OH)_3_ and −5.30 eV for In(OH)_x_S_y_‐1.0), which catalyse CO_2_ reduction to CH_4_. In contrast, the endothermic process (6.17 eV for In(OH)_3_ and 0.30 eV for In(OH)_x_S_y_‐1.0), which catalyses the reduction of CO_2_ to CO, has higher energy requirements. This indicates that both samples are more inclined toward catalysing the reduction of CO_2_ to CH_4_ rather than forming the intermediate CO. In the CO_2_ reduction pathway on the surface of the In(OH)_x_S_y_‐1.0, the energy barriers of the further adsorption of CO_2_ molecule onto the adsorbed H^+^ surface (ΔG = 0.57 eV) and the process for adsorbed CO_2_ molecule (^*^CO_2_) converting into ^*^COOH (ΔG = −0.47 eV) is higher than the CO_2_ adsorbed on the surface of the In(OH)_3_ (^*^CO_2_ with ΔG = −2.16 eV and ^*^CHO with −4.84 eV). There is no significant difference in the energy requirement for ^*^COOH converting into ^*^CO on the surface of the In(OH)_x_S_y_‐1.0 (ΔG = −3.63 eV) and the energy barrier required for that on the surface of In(OH)_3_ sample (ΔG = −5.23 eV). During the subsequent activation of CO_2_ reduction on the surface of the In(OH)_x_S_y_‐1.0, the reaction state energies in the formation of key intermediates (^*^CHO with ΔG = −2.57 eV and ^*^CH_3_O with ΔG = −1.00 eV) are lower than those of In(OH)_3_ (^*^CHO with ΔG = −1.34 eV and ^*^CH_3_O with ΔG = −0.52 eV). Although the formation energy of ^*^CH_4_ on the In(OH)_x_S_y_‐1.0 surface (ΔG = −5.00 eV) is slightly higher than that on the In(OH)_x_S_y_‐1.0 surface (ΔG = −5.22 eV), the energy required for desorption and the subsequent formation of free CH_4_ molecule is significantly lower on the In(OH)_x_S_y_‐1.0 surface (ΔG = −0.31 eV) compared to the In(OH)_3_ surface (ΔG = 3.24 eV). Moreover, considering that formic acid (HCOOH) is also a possible product in the CO_2_ photoreduction, the ^*^HCOOH formation energy and the desorption energy of ^*^HCOOH to form HCOOH molecule on the In(OH)_x_S_y_‐1.0 were further calculated. As shown in Figure S16 (Supporting Information), despite the formation energy of ^*^HCOOH (ΔG = −4.45 eV) is slightly lower than ^*^CO (ΔG = −3.63 eV), the energy barrier required for the desorption of ^*^HCOOH to form HCOOH molecule (ΔG = 0.244 eV) is much higher than that for the formation of the CH_4_ intermediate ^*^CHO (ΔG = −2.57 eV). All these calculated results indicate a more favourable reaction pathway for CO_2_‐to‐CH_4_ conversion on the In(OH)_x_S_y_‐1.0 surface.

## 3 Conclusion

In summary, a series of In(OH)_x_S_y_‐*z* nanocube catalysts prepared via the solvothermal method exhibit excellent efficiency in selectively reducing CO_2_ to CH_4_ under UV–vis light. The CH_4_ product yield of the In(OH)_x_S_y_‐1.0 catalyst (2.75 µmol g^−1^ h^−1^) is enhanced by ≈39 times compared to In(OH)_3_ (0.07 µmol g^−1^ h^−1^). Moreover, the product selectivity of CH_4_ can be enhanced ranging from 50.0% for In(OH)_3_ to 80.75% for In(OH)_x_S_y_‐1.0. This enhancement in CH_4_ production and selectivity is attributed to the “One stone three birds strategy”: 1) the substitution of sulfur atoms narrows the band gap and improves the light absorption and the separation efficiency of photoinduced carriers; 2) the introduced S site enhances hydrophilicity of In(OH)_x_S_y_‐1.0 sample and reduces H_2_O dissociation energy barrier and forms active S‐H site, promoting the generation and the transformation of protons for CO_2_ hydrogenation; 3) the presence of S^2−^ effectively inhibits the formation of strong oxidized ^•^OH radical, thereby stabilizing intermediate ^*^CHO species and facilitating wanted CH_4_ production. This study enriches the understanding of the structure‐activity relationship in photocatalysts and showcases the potential of non‐metallic doping engineering to enhance the selectivity of CO_2_ reduction by effectively modulating catalyst properties.

## 4 Experimental Section

### Materials

Indium nitrate hydrate (In(NO_3_)_3_·*x*H_2_O, ≥99.9%) was purchased from Aladdin, while thiourea (CH_4_N_2_S, AR), sodium sulfate (Na_2_SO_4_, ≥99.0%), potassium bicarbonate (KHCO_3_, ≥99.5%), ethylenediamine (EDA, ≥99.0%) and ethanol (CH_3_OH, ≥99.7%) were purchased from Sinopharm Chemical Reagent Co., Ltd. All of the chemical reagents were used without further purification. High‐purity CO_2_ (≥99.999%) and high‐purity nitrogen (N_2_, ≥99.999%) were provided by Jining Xieli Special Gas Co., Ltd. Additionally, throughout the entire experimental process, the deionized (DI) water used was self‐prepared using the laboratory's ultrapure water system.

### Preparations of Photocatalysts

A series of In(OH)_x_S_y_ *‐z* with different S/In atomic ratios were synthesized by hydrothermal method as previous report.^[^ ^16b^ ^]^ In a typical synthesis, In(NO_3_)_3_·*x*H_2_O (1.5 mmol) and the desired amount of thiourea (with S/In atomic ratios of 0, 0.25, 0.5, 1.0, 1.5, and 2.0) were dissolved in a mixture of 1 mL EDA and 14 mL DI water. After stirring for 30 min, the above solution was transferred to a 25 mL Teflon‐lined stainless‐steel autoclave and heated at 180 °C for 20 h. The as‐prepared products were collected by centrifugation and washed several times with DI water and ethanol. After vacuum drying at 60 °C for 12 h and grinding, the yellow powders obtained were labelled as In(OH)_x_S_y_‐*z* samples, where *z* referred to the S/In atomic ratios. When the S/In atomic ratio was 0, it was labelled as In(OH)_3_ sample.

### Characterizations

Power X‐ray diffraction (XRD) patterns were recorded on a Miniflex600 diffractometer (Rigaku, Japan) with Cu Kα radiation (*λ* = 0.15418 nm). Transmission electron microscope (TEM) and high‐resolution transmission electron microscope (HRTEM) images were examined on a JEM‐2100 microscope (Japan) at 200 kV. UV–vis diffused reflectance spectra (UV–vis DRS) were tested on a Shimadzu UV‐2600i spectrophotometer equipped with an integrating sphere attachment in the wavelength range of 220–800 nm. The Brunauer–Emmet–Teller (BET) adsorption and desorption isotherm of N_2_ and CO_2_ were determined on a Kubo X1000 BET analyzer (China) at 77 and 298 K, respectively. The water contact angle pictures were recorded on an optical contact angle meter with SL250 (Kino, USA) after subjecting the samples to prepressing at 8 MPa. The Raman spectrums were characterized on a LabRAM HR Evolution Raman spectrometer (Horiba Scientific, France) by using a 532 nm laser beam. X‐ray photoelectron spectra (XPS) measurements were performed on an ESCALAB 250 photoelectron spectroscopy (Thermo Fisher Scientific, USA) at 3.0 × 10^−10^ mbar with monochromatic Al Kα Radiation. The electron paramagnetic resonance (EPR) spectra were collected using a Bruker model A300 Paramagnetic spectrometer (German). CO temperature programmed desorption (CO‐TPD) measurements were operated on an AMI‐300 chemisorption analyzer (Altamira Instruments, USA). Room temperature photoluminescence (PL) spectra were obtained from an FS5 photoluminescence spectroscopy (Edinburgh Instruments, UK) with a 248 nm excitation light source. The relaxation of excited states through radiative or nonradiative recombination processes were investigated using femtosecond transient absorption (fs‐TA) spectroscopy with an Ultrafast Systems Helios instrument (USA). The ζ potentials were collected on a Malvern Zetasizer Nano ZS system (Malvern Panalytical, UK) in an aqueous solution. In situ diffuse reflectance infrared Fourier transform spectra (DRIFTS) were performed on a Nicolet 670 FT‐IR (Thermo Nicolet, USA) spectrometer (Bruker, Germany).

### EXAFS Measurements

The In K‐edge analysis was performed with Si (311) crystal monochromators at the BL11B beamlines at the Shanghai Synchrotron Radiation Facility (SSRF) (Shanghai, China). Before the analysis at the beamline, samples were pressed into thin sheets with 1 cm in diameter and sealed using Kapton tape film. The XAFS spectra were recorded at room temperature using a 4‐channel Silicon Drift Detector (SDD) Bruker 5040. In K‐edge extended X‐ray absorption fine structure (EXAFS) spectra were recorded in transmission mode. Negligible changes in the line‐shape and peak position of In K‐edge XANES spectra were observed between two scans taken for a specific sample. The XAFS spectra of these standard samples (In foil, In_2_O_3_, In(OH)_3_ and In(OH)_x_S_y_‐1.0) were recorded in transmission mode. The spectra were processed and analyzed by the software codes Athena and Artemis.

### Photoelectrochemical Tests

Mott–Schottky (M–S) analysis, transient photocurrent response, cyclic voltammetry (CV) curves and the oxidation overpotential of H_2_O were conducted on a CHI660E electrochemical analyzer (CH Instrument, USA) in 0.5 m Na_2_SO_4_ electrolyte. For the transient photocurrent response the light comes from a 300 W Xe lamp (CEL‐PF300‐T8, Beijing China Education Au‐Light, China), and for CV curves the scan rate was 2 mV s^−1^. Linear sweep voltammetry (LSV) curves of CO_2_ reduction were measured in a CO_2_‐saturated 0.5 m KHCO_3_ electrolyte at a scan rate of 2 mV s^−1^ in an H‐style cell on a CHI660E with a 300W Xe lamp. Electrochemical impedance spectrum (EIS) measurements were conducted on an Autolab 302A electrochemical analyzer (Metrohm, Switzerland) in 0.5 m Na_2_SO_4_ electrolyte. An Ag/AgCl electrode and Pt foil served as the reference and counter electrode, respectively, in the entire photoelectrochemical procedures.

### Photocatalytic Performance

Photocatalytic reduction of CO_2_ with H_2_O was performed in a 25 mL custom‐made glass diagonal bipass reaction tube with an extra passage. 15 mg photocatalyst was first dispersed in 15 mL DI water. The reactor was then evacuated and refilled with CO_2_ five times to remove the air inside. Finally, the reactor was filled with CO_2_ until the pressure reached 0.1 MPa. During the photoreduction, the reactor was irradiated by an Xe lamp (CEL‐PF300‐T8, 300 mW cm^−2^, Beijing China Education Au‐Light, China) under vigorous stirring. The temperature of the reactor was detected by using a kerosene thermometer and was maintained at ≈80 °C arising from the Xenon lamp illumination. The products were analyzed on gas chromatography (GC‐7920, Beijing China Education Au‐Light, China) equipped with a flame ionization detector (FID) and a thermal conductivity detector (TCD).

### Theoretical Calculation Methods

The structure models were performed according to the first‐principles by using the density functional theory (DFT), as implemented in the Cambridge Sequential Total Energy Package (CASTEP) computational codes. During the geometry optimization of In(OH)_3_ and In(OH)_x_S_y_‐1.0, lattice parameters and atomic positions were optimized simultaneously. The model of In(OH)_x_S_y_‐1.0 was established by replacing two hydroxyls with two S^2−^ on the crystal (200) plane of In(OH)_3_. For calculating the electronic structures and density of states, the geometry optimization of crystal (200) plane of In(OH)_3_ and In(OH)_x_S_y_‐1.0 were calculated by the PBE method within Generalized Gradient‐corrected Approximation (GGA) with the exchange‐correlation potential. The Vanderbilt ultrasoft pseudopotential with a cutoff energy of 340 eV was used to ensure the precision of the results. Brillouin zone integration was performed using the *Monkhorst–Pack* scheme with a 3 × 3 × 1 K‐point sampling scheme. The convergence tolerance for geometry optimization was selected with the differences in total energy (1.0 × 10^−5^ eV per atom), the maximal ionic *Hellmsann‐Feynman* force (3.0 × 10^−2^ eV Å^−1^), the stress tensor (5.0 × 10^−2^ GPa), and the maximal displacement (1.0 × 10^−3^ Å).

### The Equations Used for Calculations

The average crystallite size was calculated using the *Debye–Scherrer* formula, defined as the Equation (1):

(1) $$D\;=\frac{K\cdot \lambda }{\beta \cdot \cos\theta }\;$$

where *D* represents the average crystallite size of samples, λ is the wavelength of the X‐ray (with a value of 1.542 Å due to the use of X‐ray copper tubes), *β* is the full‐width half maximum peak, and θ is *Braggs’* angle. The values of *K* are ≈0.94 for bulk and spherical‐shaped particles.

The values of band gap were calculated using the *Kubelka–Munk* function, defined as the Equations (2), (3), and (4):

(2) $$F\;\left(R\right)=\frac{{\left(1-R\right)}^{2}}{2R}\;$$

(3) $$h\cdot \nu \;=\frac{1240}{\lambda }\;$$

(4) $$E_{g}={\left(F\left(R\right)\cdot h\cdot \nu \right)}^{n}\;$$

where *F*(*R*) represents the absorption intensity, *R* is the reflectivity of samples, *h* is the Planck constant, ν is the frequency of the incident light, and λ is the wavelength of the incident light. *E_g_* is the value of the band gap. The value of the exponent *n* depends on the type of semiconductor. In the case of In(OH)_3_, which is a direct bandgap semiconductor, the value of *n* is 1/2.

The conduction band values were calculated using the *Nernst* equation in comparison to the normal hydrogen electrode as reported,^[^ ^21^ ^]^ defined as the Equation (5):

(5) $$E_{NHE}=E_{Ag/Agcl}\;+0.1976\;V$$

where *E_NHE_* represents the values of the conduction band of samples in comparison to the normal hydrogen electrode, and *E*_*Ag*/*Agcl*_ is the value of the reference electrode used.

The reduction potentials were calculated using the *Nernst* equation in comparison to the reversible hydrogen electrode as reported,^[^ ^39^ ^]^ which is dependent on the *pH* of the solution and is defined as Equation (6):

(6) $$E_{RHE}=E_{Ag/Agcl}\;+0.059\;V\cdot pH+0.196\;V$$

where *E_RHE_* represents the values of the reduction potential of samples in comparison to the reversible hydrogen electrode, *E*_*Ag*/*Agcl*_ is the value of the reference electrode used. The *pH* represents the hydrogen ion concentration in an electrolyte solution. In the 0.5 m KHCO_3_ electrolyte, the *pH* is 7.21.

The product selectivity and electron selectivity of reduction productions was deduced according to the reported equation,^[^ ^44b^ ^]^ defined as the Equations (7) and (8):

(7) $$Production\;Selectivity_{\left(CH_{4}\right)}=\frac{R_{CH_{4}}}{R_{CH_{4}}+R_{CO}}\;\times 100\%$$

(8) $$Electron\;Selectivity_{\left(CH_{4}\right)}=\frac{8R_{CH_{4}}}{8R_{CH_{4}}+2R_{CO}}\;\times 100\%$$

where $Production\;Selectivity_{\left(CH_{4}\right)}$ represents the product selectivity values of CH_4_, $Electron\;Selectivity_{\left(CH_{4}\right)}$ represents the electron selectivity values of CH_4_. The $R_{CH_{4}}$ and *R_CO_* represent the production rate of CH_4_, and CO respectively.

## Conflict of Interest

The authors declare no conflict of interest.

## Supporting information

Supporting Information

## Data Availability

The data that support the findings of this study are available from the corresponding author upon reasonable request.
