# Supplementary material for: Non‐Metal Sulfur Doping of Indium Hydroxide Nanocube for Selectively Photocatalytic Reduction of CO2 to CH4: A “One Stone Three Birds” Strategy
Source: Adv Sci (Weinh). 2024 Jun 13;11(30):2401990. doi: 10.1002/advs.202401990 (PMC11321682; doi:10.1002/advs.202401990)
Supplement: Supplementary file 1 — Supporting Information [file ADVS-11-2401990-s001.pdf]

ADVANCED SCIENCE
Open Access

Supporting Information

for *Adv. Sci.*, DOI 10.1002/advs.202401990

Non-Metal Sulfur Doping of Indium Hydroxide Nanocube for Selectively Photocatalytic Reduction of CO_2 to CH_4: A “One Stone Three Birds” Strategy

*Qinhui Guan, Weiguang Ran, Dapeng Zhang, Wenjuan Li, Na Li, Baibiao Huang and Tingjiang Yan**

Supporting Information

Non-metal sulfur doping of indium hydroxide nanocube for selectively photocatalytic reduction of CO_2 to CH_4: A "one stone three birds" strategy

Qinhui Guan,[a] *Weiguang Ran,*[b] *Dapeng Zhang,*[b] *Wenjuan Li,*[b] *Na Li,*[c] *Biabiao Huang,*[c] *Tingjiang Yan*[a,b]*

[a] College of Chemistry and Chemical Engineering, Shaanxi University of Science and Technology, Xi'an 710021, P.R. China

[b] Key Laboratory of Catalytic Conversion and Clean Energy in Universities of Shandong Province, School of Chemistry and Chemical Engineering, Qufu Normal University, Qufu 273165, P.R. China

[c] State Key Laboratory of Crystal Materials, Shandong University, Jinan 250100, P.R. China

Corresponding author Tel & Fax: (+86)537-4458301, E-mail: tingjiangn@163.com

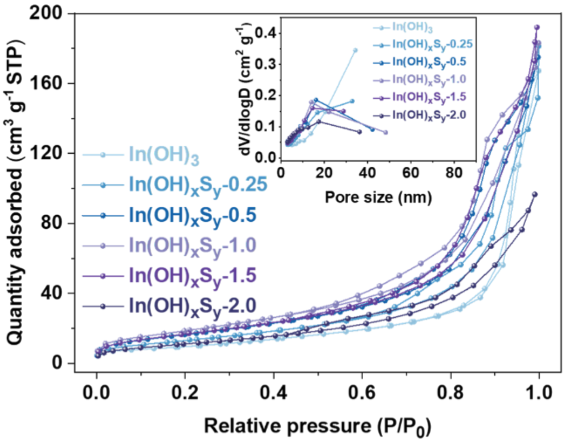

Figure S1 BET diagrams and pore size distribution charts (inset) of $In(OH)_3$ and $In(OH)_xS_y$-*z* samples.

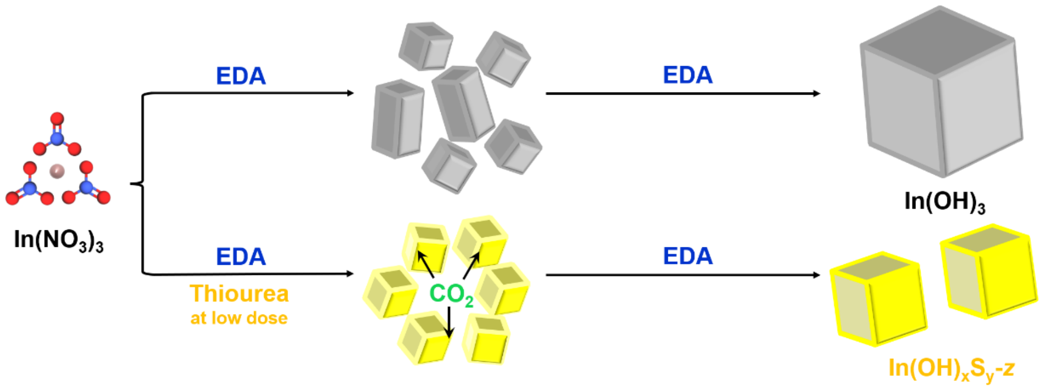

Figure S2 Possible formation mechanism of $In(OH)_3$ and $In(OH)_xS_y$-*z* nanocubes.

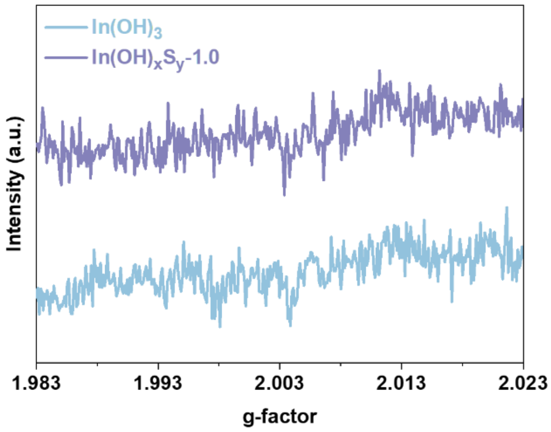

Figure S3 EPR curves of $In(OH)_3$ and $In(OH)_xS_y$-1.0.

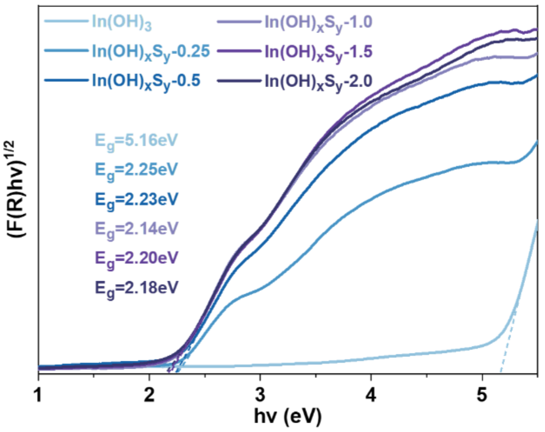

Figure S4 The band-gap of $In(OH)_3$ and $In(OH)_xS_y$-*z* samples.

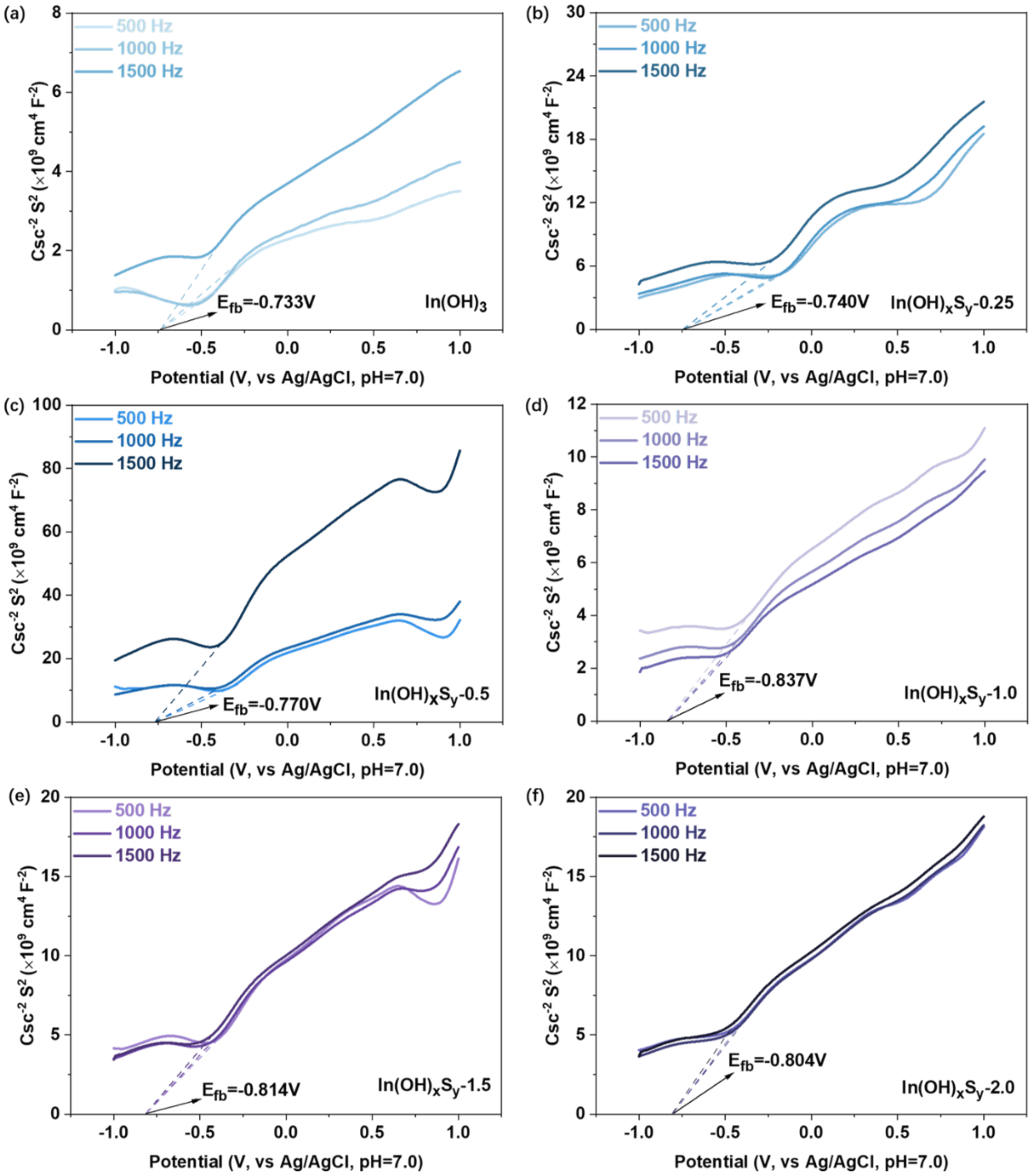

Figure S5 *Mott−Schottky* plots of (a) $In(OH)_3$, (b) $In(OH)_xS_y$-0.25, (c) $In(OH)_xS_y$-0.5, (d) $In(OH)_xS_y$-1.0, (e) $In(OH)_xS_y$-1.5 and (f) $In(OH)_xS_y$-2.0.

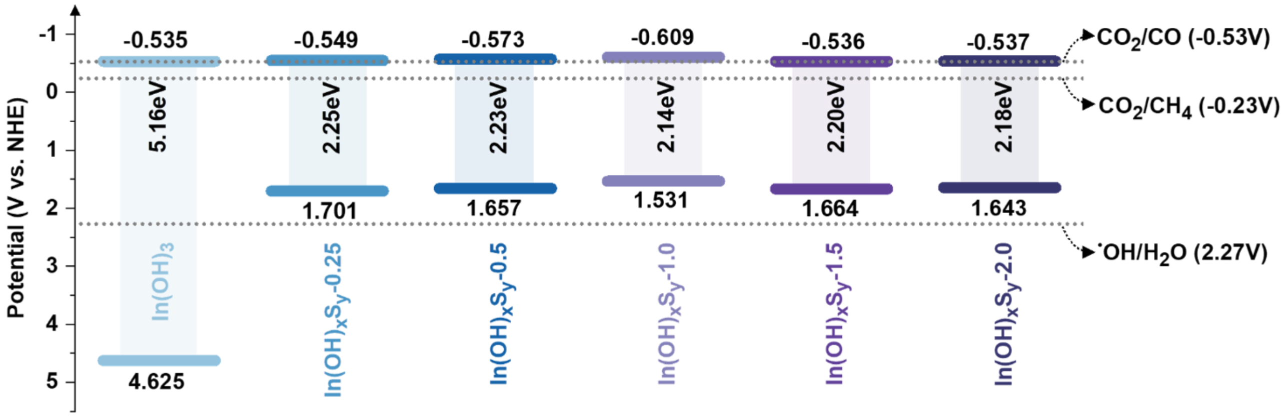

Figure S6 The diagram of the electronic band structure of $In(OH)_3$ and $In(OH)_xS_y$-*z* samples.

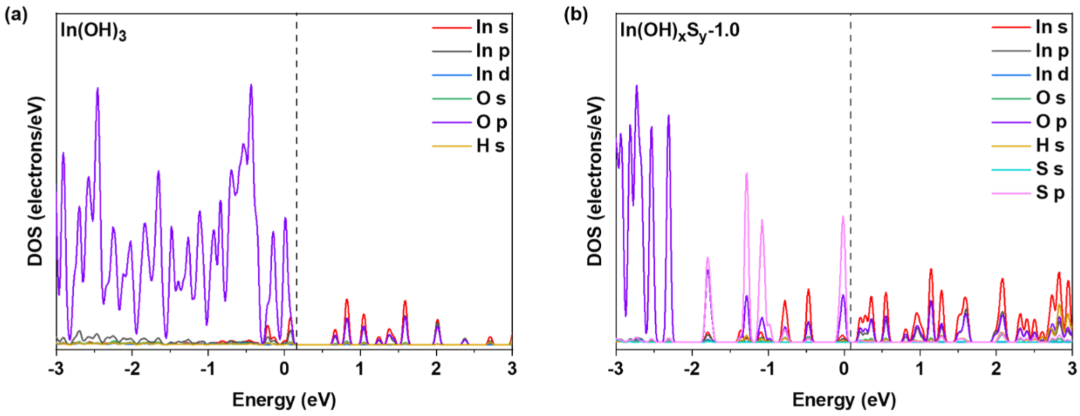

Figure S7 Partial density of states (PDOS) of (a) $In(OH)_3$ and (b) $In(OH)_xS_y$-1.0, respectively.

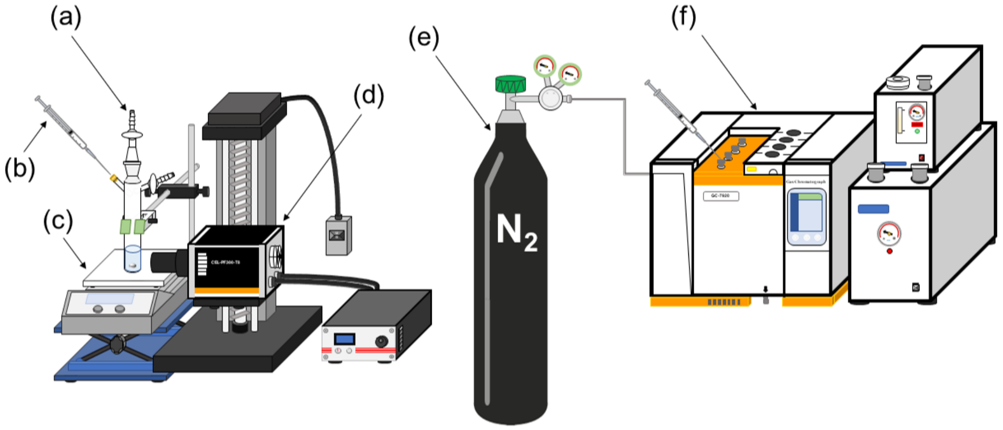

Figure S8 Detailed configuration of Photocatalytic CO_2 reduction tests. (a) Glass diagonal bipass reaction tube with an extra passage; (b) sampling needle; (c) magnetic stirrer; (d) Xenon lamp; (e) high purity N_2 carrier gas; (f) gas chromatography.

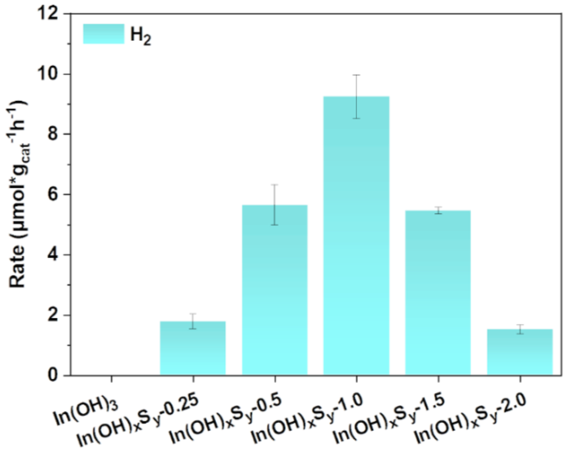

Figure S9 Production rate of H_2 over $In(OH)_3$ sample and $In(OH)_xS_y$-*z* sample.

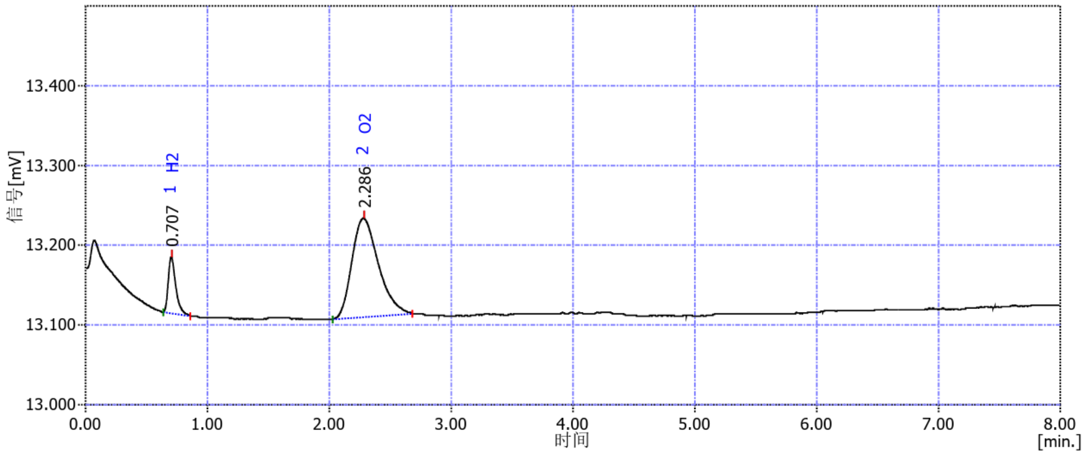

Figure S10 TCD signal in the gas chromatograph during the CO_2 photoreduction.

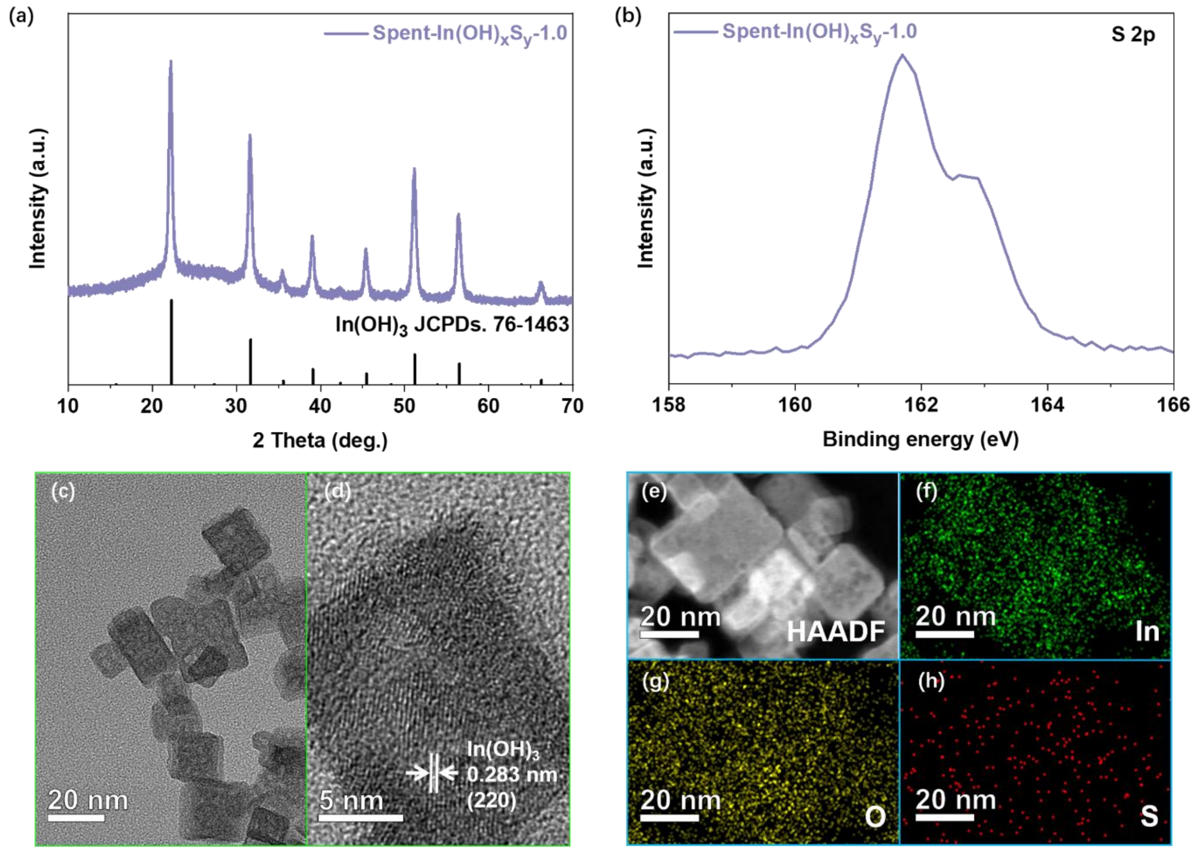

Figure S11 The (a) XRD, (b) high resolution XPS of S 2p, (c-d) TEM images and (e-f) EDS element mapping images of the spent-$In(OH)_xS_y$-1.0 after 5 successive cycle tests.

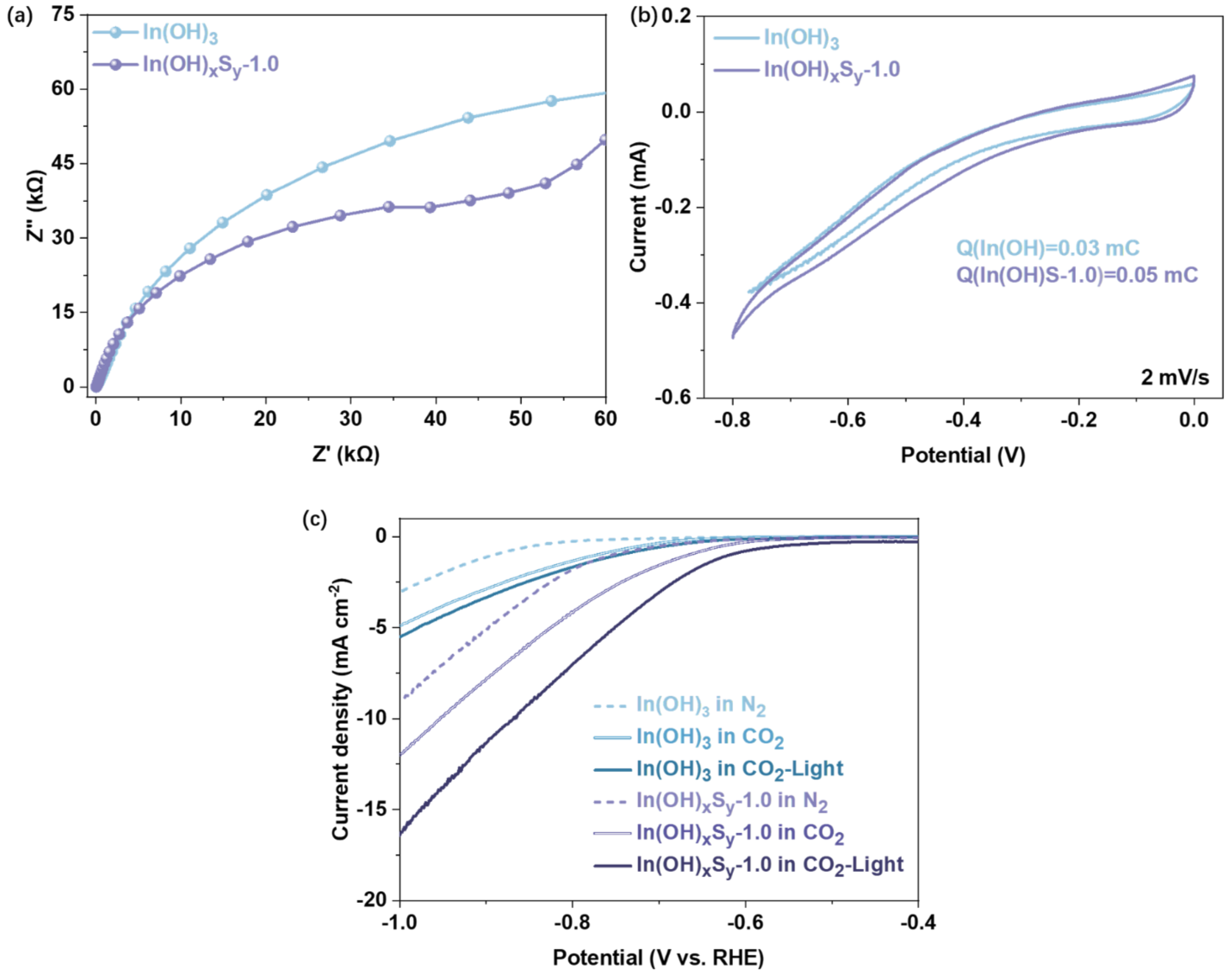

Figure S12 Photochemical and photo-electrocatalytic measurements. The (a) CV curves, (b) EIS spectra and (c) LSV curves in N_2-saturated atmosphere, CO_2-saturated atmosphere and CO_2-saturated atmosphere with light irradiation of $In(OH)_3$ sample and $In(OH)_xS_y$-1.0 sample.

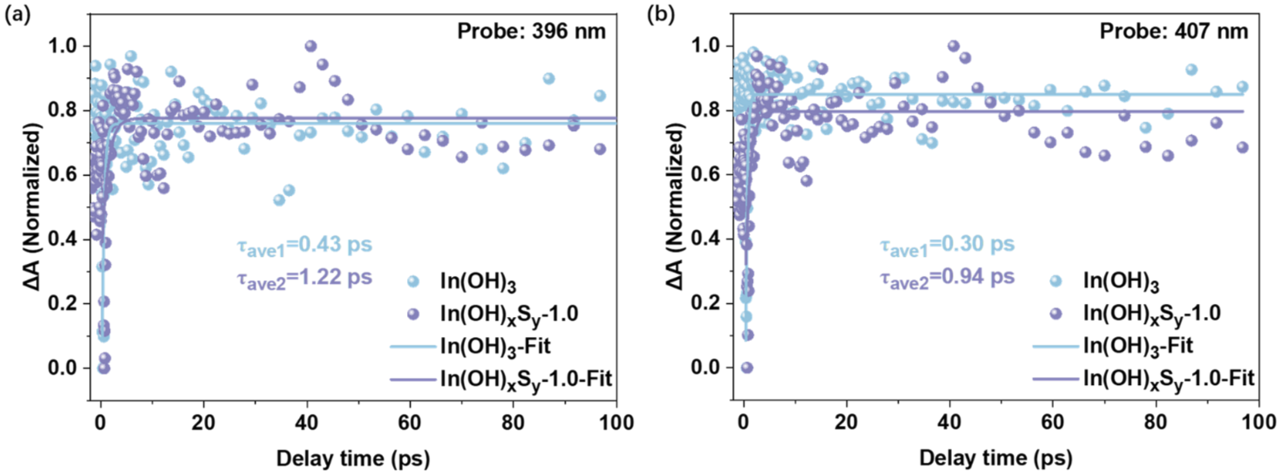

Figure S13 The fs-TA kinetic decay plots and corresponding fitting curves of $In(OH)_3$ and $In(OH)_xS_y$-1.0 at (a) 396 nm and (b) 407 nm.

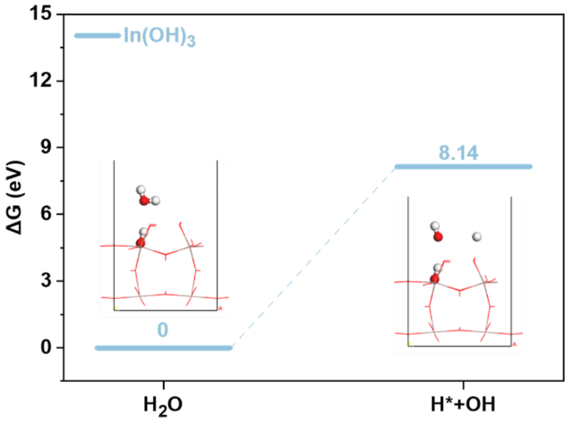

Figure S14 DFT-calculated Gibbs free energy diagrams of H_2O activation and dissociation on the surfaces of $In(OH)_3$ sample.

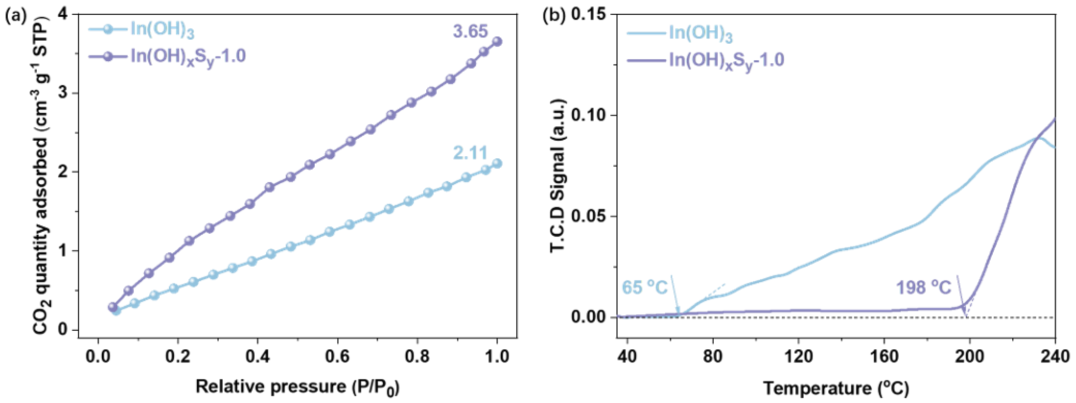

Figure S15 Adsorption abilities for CO_2 and CO. (a) CO_2 adsorption isotherms and (b) CO-TPD of $In(OH)_3$ sample and $In(OH)_xS_y$-1.0 sample.

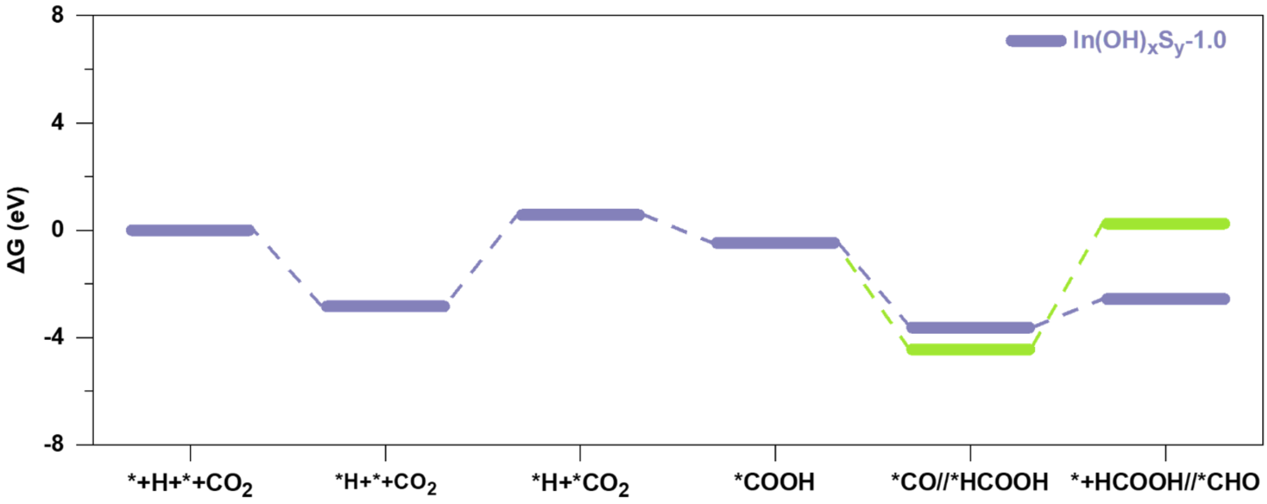

Figure S16 Gibbs free energy diagrams of reaction pathways. Photocatalytic CO_2 reduction on the surfaces of In(OH)$_x$S$_y$-1.0. The lilac lines refer to the pathway of CO* and CHO* formation, while the pale green lines refer to the pathway of *HCOOH and HCOOH formation.

Table S1 Comparison of corresponding microscopic parameters of different substances.

Sample	Crystallite size[a] D_{XRD}(nm)	Surface area[b] (m^2 g^{-1})	Mean pore size[b] (nm)	Zeta potential (mV)
$In(OH)_3$	27.35	35.69	3.18	4.67
$In(OH)_xS_y$-0.25	23.51	47.18	2.94	-14.61
$In(OH)_xS_y$-0.5	20.10	62.39	3.00	-16.64
$In(OH)_xS_y$-1.0	19.88	69.71	3.09	-17.37
$In(OH)_xS_y$-1.5	19.88	63.06	2.96	-16.07
$In(OH)_xS_y$-2.0	19.69	41.86	2.88	-15.81

[a] Crystallite size from *Debye Scherrer* equation.

[b] Obtained from BET method.

Table S2 Comparison of corresponding microscopic parameters of different samples.

Sample	Relative atomic ratio[a] (%)			The actual chemical formula
	In	O	S	
$In(OH)_3$	23.18	76.82	0	$In(OH)_3$
$In(OH)_xS_y$-1.0	24.91	70.03	5.06	$In(OH)_{2.8}S_{0.2}$

[a] Obtained from XPS data.

The chemical formulas derived from the XPS results align with previously reported literature.[1]

Table S3 EXAFS fitting parameters at the In K-edge for various samples.

Sample	Shell	CN^{a}	R (Å)[b]	σ^2 (Å^2)[c]	ΔE_0 (eV)[d]	R factor
In foil	In-In	12*	3.291±0.011	0.0140	-1.2	0.0098
In_2O_3	In-O	3.1±0.4	2.112±0.015	0.0024	2.6	0.0056
	In-O	3.0±0.3	2.218±0.016	0.0024		
	In-O-In	6.2±0.6	3.345±0.003	0.0051		
	In-O-In	5.7±0.4	3.836±0.005	0.0071		
$In(OH)_3$	In-O	6.0±0.2	2.163±0.006	0.0052	3.7	0.0070
$In(OH)_xS_y$-1.0	In-O	4.9±0.2	2.101±0.008	0.0054	0.0070	0.0031
	In-S	0.9±0.3	2.364±0.025	0.0054	0.0031	

[a] *CN*, coordination number.

[b] *R*, the distance to the neighboring atom.

[c] σ^2, the Mean Square Relative Displacement (MSRD).

[d] ΔE_0, inner potential correction; *R* factor indicates the goodness of the fit.

* This value was fixed during EXAFS fitting, based on the known structure of In.

$S0^2$ was fixed to 0.815, according to the experimental EXAFS fit of In foil by fixing *CN* as the known crystallographic value. Fitting range: $2.0 \leq k$ (/Å) ≤ 12.5 and $1.1 \leq R$ (Å) ≤ 2.3 ($In(OH)_3$ and $In(OH)_xS_y$-1.0). A reasonable range of EXAFS fitting parameters: $0.700 < S_0^2 < 1.000$; $CN > 0$; $\sigma^2 > 0$ Å^2; $|\Delta E_0| < 15$ eV; R factor < 0.02.

Data reduction, data analysis, and EXAFS fitting were performed and analyzed with the Athena and Artemis programs of the Demeter data analysis packages that utilizes the FEFF6 program to fit the EXAFS data.[2] The energy calibration of the sample was conducted through standard and In foil, which as a reference was simultaneously measured. A linear function was subtracted from the pre-edge region, then the edge jump was normalized using Athena software. The χ(k) data were isolated by subtracting a smooth, third-order polynomial approximating the absorption background of an isolated atom. The k^3-weighted $\chi(k)$ data were Fourier transformed after applying a *HanFeng* window function ($\Delta k = 1.0$). For EXAFS modeling, The global amplitude EXAFS (CN, R, σ^2 and ΔE_0) were obtained by nonlinear fitting, with least-squares refinement, of the EXAFS equation to the Fourier-transformed data in *R*-space, using Artemis software, EXAFS of the In foil are fitted and the obtained amplitude reduction factor S_0^2 value (0.815) was set in the EXAFS analysis to determine the coordination numbers (*CNs*) in the In-O and In-S scattering path in sample.

Table S4 Comparison of the CH_4 yield and selectivity of $In(OH)_xS_y$-*z* with recently reported $In(OH)_3$-based photocatalysts.

Number	Sample	The yield of CH_4 ($\mu mol\ g^{-1}\ h^{-1}$)	The selectivity of CH_4 (%)	Reference
1	$In(OH)_xS_y$-1.0	2.75	80.75	This work
2	$In(OH)_3$	0.07	50.05	This work
3	1.5%La-$In(OH)_3$	56.56	61.97	[3]
4	4/1-$In(OH)_3$	28.20	73.44	[4]
5	mesoporous-$In(OH)_3$	0.80	100	[5]
6	$ZnIn_2S_4/In(OH)_{3-x}$	0 (Only produced CO)	0	[6]
7	Bi-doped $In(OH)_3$	0 (Only produced CO)	0	[7]
8	ZnS-$In(OH)_3$ 7%	0 (Only produced CO)	0	[8]
9	TP/$In(OH)_3$	0 (Only produced CO)	0	[9]

References

[1] Z. Lei, G. Ma, M. Liu, T. Takata, W. You, M. Hara, H. Yan, K. Domen, G. Wu, CanLi, *J. Catal.* **2006**, *237*, 322.

[2] a) B. Ravel, M. Newville, *J. Synchrotron Radiat.* **2005**, *12*, 537; b) J. J. R. S. I. Zabinsky, A. Ankudinov, R. C. Albers, M. J. Eller, *Phys. Rev. B* **1995**, *52*, 2995.

[3] Z. Wan, M. Hu, B. Hu, T. Yan, K. Wang, X. Wang, *Catal. Sci. Technol.* **2020**, *10*, 2893.

[4] B. Hu, M. Hu, Q. Guo, K. Wang, X. Wang, *Appl. Catal. B* **2019**, *253*, 77.

[5] J. Guo, S. Ouyang, T. Kako, J. Ye, *Appl. Surf. Sci.* **2013**, *280*, 418.

[6] X. Liang, X. Wang, X. Zhang, S. Lin, M. Ji, M. Wang, *ACS Catal.* **2023**, *13*, 6214.

[7] C. Liu, Q. Zhu, Z. Zhu, C. Sun, Y. Xuan, K. Zhang, *J. Solid State Chem.* **2022**, *311*, 123141.

[8] Q. Zhao, H. Li, Y. Cao, *J. Solid State Chem.* **2021**, *296*, 121976.

[9] M. C. Hsieh, G. C. Wu, W. sG. Liu, W. A. G. III, C. M. Yang, *Angew. Chem. Int. Ed.* **2014**, *53*, 14216.
